# Sex as a Contextual Modifier in Colorectal Cancer: Integrating Tumor Sidedness, Molecular Subtype, Immune Ecology, and Early-Onset Disease

**DOI:** 10.3390/cancers18081309

**Published:** 2026-04-21

**Authors:** Bing Liang, Xinlin Liu, Tingting Zhang, Dongming Xing

**Affiliations:** 1 Cancer Institute, The Affiliated Hospital of Qingdao University, Qingdao University, Qingdao 266000, China; lxl2021910024@qdu.edu.cn (X.L.); zhangtt@qdu.edu.cn (T.Z.); xingdongming@qdu.edu.cn (D.X.); 2 School of Life Sciences, Tsinghua University, Beijing 100084, China

**Keywords:** colorectal cancer, sex as a biological variable, tumor sidedness, immune microenvironment, molecular subtype, early-onset colorectal cancer

## Abstract

Colorectal cancer (CRC) shows consistent sex-related differences in incidence, anatomic distribution, molecular subtype, immune context, and clinical outcome. However, these differences are often discussed through broad parallel themes such as hormones, genetics, or the microbiome, rather than through the biological settings in which sex meaningfully modifies tumor behavior. This review argues that sex is most informative in CRC when treated as a contextual modifier whose relevance emerges only after integrating tumor sidedness, mismatch repair status, oncogenic background, immune ecology, and age at onset. The clearest signals arise from interaction-based contexts, particularly when sex is interpreted together with tumor sidedness and dMMR/MSI-H or *BRAF*-linked disease states. Current evidence indicates that women are enriched for proximal or right-sided, microsatellite instability-high, mismatch repair-deficient, CpG island methylator phenotype-high, and *BRAF*-associated CRC, whereas men more often present with distal disease and a higher overall burden. Mechanistic studies further show that sex-related differences extend beyond hormone exposure to include *KRAS*–*STAT4*–*KDM5D* signaling, site-specific immune-checkpoint programs, metabolic phenotypes, epigenetic biomarker variation, and microbiota–hormone crosstalk. These effects are most evident in defined clinical niches, particularly right-sided CRC, mismatch repair-deficient disease, *BRAF*-mutated metastatic CRC, and early-onset CRC. A sex-aware, subtype-aware, and location-aware framework therefore offers a more clinically useful interpretation of CRC heterogeneity than descriptive male-versus-female comparisons alone.

## Simple Summary

Sex-related differences in colorectal cancer are well recognized, but they are often described too broadly to guide biological interpretation or clinical use. Here, sex is treated as most informative when interpreted together with tumor location, molecular subtype, immune context, and age at onset. The strongest sex-associated signals cluster in selected settings, including right-sided disease, mismatch repair-deficient and microsatellite instability-high tumors, *BRAF*-associated metastatic colorectal cancer, and early-onset colorectal cancer. Hormone signaling, microbial ecology, metabolism, and immune programs remain relevant, but their effects appear context dependent rather than universal. Viewing sex as a contextual modifier rather than a stand-alone binary variable offers a more precise framework for biomarker development, prognostic modeling, and future trial stratification.

## 1. Introduction

Colorectal cancer (CRC) remains a major global health burden. GLOBOCAN 2022 estimates place CRC among the most frequently diagnosed cancers worldwide and among the leading causes of cancer death, while the most recent United States cancer statistics show that an estimated 158,850 new CRC cases and 55,230 CRC deaths will occur in the United States in 2026, and that CRC is now the second most common cause of cancer-related death overall and the leading cause of cancer-related death in adults younger than 50 years in that country [1,2]. Contemporary CRC management has also become increasingly dependent on molecularly informed risk stratification and treatment selection, particularly in advanced disease [3].

These epidemiological observations are directly relevant to the present review because sex-associated differences are evident at multiple levels of CRC burden. Men consistently have higher incidence and mortality rates [4,5,6,7,8,9]. By contrast, women are more often diagnosed with proximal or right-sided tumors and are relatively enriched for selected molecular contexts, including deficient mismatch repair (dMMR), microsatellite instability-high (MSI-H), CpG island methylator phenotype-high (CIMP-high), and *BRAF*-associated disease, especially in right-sided colon cancer [4,7,8,9,10,11,12]. Yet sex is still often handled as a background demographic covariate rather than as a biologically informative stratification variable.

A second source of confusion is the frequent conflation of sex and gender. Sex refers to biological attributes, including chromosomes, reproductive anatomy, and hormone milieu; gender refers to sociobehavioral constructs that can influence risk exposure, screening uptake, diagnostic interval, treatment allocation, toxicity reporting, and survivorship [4,5,6,7,8]. Direct CRC studies that formally model sex and gender simultaneously remain limited, in part because most clinical and registry datasets still record sex more reliably than gender-related variables. Nevertheless, contemporary reviews and population-based analyses indicate that gender-shaped processes influence screening uptake, route to diagnosis, treatment allocation, survival, and time to treatment, whereas mechanistic tumor biology is more appropriately discussed within sex-aware analyses [4,8,13,14]. Accordingly, tumor biology is discussed primarily under sex in this review, whereas pathways of care, survivorship, and exposure-related behaviors are highlighted as domains in which gender should also be measured and modeled.

The central limitation of both the earlier manuscript and much of the existing literature is structural rather than purely factual. Sex-related differences in CRC are often reviewed as three parallel narratives—genetics, estrogen signaling, and microbiota—without first identifying the biological settings in which sex meaningfully changes interpretation. This review therefore advances a more selective thesis: sex is most clinically useful when understood as a contextual modifier whose effects depend on tumor sidedness, molecular subtype, immune microenvironment, and age at onset. The key question is not whether CRCs from women and men differ in the abstract, but when, where, and through which mechanisms those differences become biologically and translationally consequential. Accordingly, descriptive epidemiology, mechanistic inference, and clinically actionable implications are treated as related but distinct levels of interpretation throughout this review.

### Review Scope and Evidence Prioritization

For transparency, the literature discussed in this narrative review was identified through focused searches of PubMed, Web of Science, and Scopus using combinations of the terms colorectal cancer, sex, gender, sidedness, mismatch repair, microsatellite instability, KRAS, BRAF, estrogen receptor, microbiome, immune microenvironment, and early-onset colorectal cancer. Searches were updated through April 2026. Priority was given to contemporary human studies, tissue-based analyses, clinically annotated cohorts, and high-impact translational papers published from 2019 onward, whereas earlier seminal studies were retained when they remained mechanistically foundational. Preclinical studies were used primarily to support biologic plausibility, whereas clinically actionable statements were preferentially anchored in human cohorts and clinically annotated datasets. Where relevant, the review specifies whether evidence derives from preclinical models, retrospective cohorts, pooled analyses, prospective tissue studies, or hypothesis-generating translational work.

## 2. From Descriptive Sex Differences to Contextual Sex Biology

Earlier work established an important descriptive foundation by documenting epidemiological and molecular differences between men and women patients with CRC. Those observations remain valuable, but descriptive comparisons alone have limited explanatory power because CRC is not a single biological entity. Right-sided and left-sided tumors differ in embryologic origin, stromal organization, mutational architecture, metabolic state, immune tone, and response to systemic therapy; similarly, MSI-H, chromosomally unstable, *KRAS*-driven, and *BRAF*-associated CRCs represent biologically distinct disease contexts [7,8,9,15,16,17].

Once this heterogeneity is acknowledged, a clearer pattern emerges: many apparent sex effects become stronger after stratification and weaker when all CRCs are pooled together. The enrichment of women in proximal tumors, MSI-related biology, and *BRAF*-mutated disease is more informative than a crude all-comer comparison, and the same is true for men-linked *KRAS*-associated programs. Conversely, the *KDM5D* program identified in men with *KRAS*-mutant colon cancer is informative precisely because it is mechanistically tied to a defined oncogenic background rather than to male sex alone [7,8,15,16,17]. In practical terms, sex behaves like an effect modifier. A prognosis model or treatment analysis that ignores sex × sidedness or sex × molecular-subtype interactions can dilute signals that become visible only inside defined biological strata. This is why right-sided dMMR/MSI-H disease, *BRAF*-associated metastatic CRC, and *KRAS*-mutant tumors are more informative test beds for sex-aware interpretation than unselected CRC cohorts.

**Figure 1:**
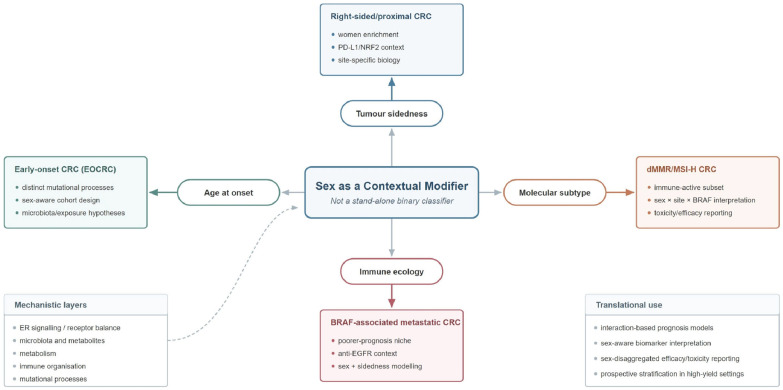
Sex as a contextual modifier in colorectal cancer. The schematic illustrates sex as an effect modifier whose clinical relevance becomes most apparent when interpreted together with four high-confidence disease contexts: right-sided/proximal tumors, dMMR/MSI-H tumors, *BRAF*-associated metastatic CRC, and EOCRC. Within each context, sex-linked effects are shaped by partially overlapping mechanistic layers, including immune organization, receptor signaling, metabolism, microbiota, and mutational processes.

This contextual approach also changes how the older estrogen and microbiota literature should be interpreted. Estrogen signaling, estrogen receptor beta (ERβ) loss, G protein-coupled estrogen receptor (GPER) activity, and the gut microbial ecosystem remain relevant in CRC. However, they are better understood as biological layers that modify selected CRC states—especially proximal disease, immune-active tumors, and early-onset CRC (EOCRC)-related ecological exposures—rather than as universal explanations for all sex-related heterogeneity in CRC. Accordingly, receptor signaling, epigenetic circuitry, microbiota, and immunity are discussed here as nested mechanistic layers within interaction-based disease contexts rather than as parallel standalone themes. This interaction-based framework is summarized in Figure 1. To improve continuity, the remainder of the review proceeds from disease context to mechanistic layer: Section 3 first defines the sidedness- and subtype-dependent settings in which sex is most informative, Section 4 then revisits ER-associated signaling within those contexts rather than as a stand-alone explanatory model, and Section 5, Section 6, Section 7 and Section 8 extend the same logic to microbiota, immune organization, biomarker/therapeutic implications, and EOCRC.

## 3. Sex, Tumor Sidedness, and Molecular Architecture

One of the most reproducible findings in this field is the non-random anatomical distribution of CRC by sex. Women are more likely to develop proximal or right-sided colon cancer, whereas men more often present with distal colon and rectal tumors and a higher overall disease burden [6,7,8,9]. Tumor sidedness is not merely descriptive. Right-sided CRC is enriched for MSI-H, CIMP-high, *BRAF* V600E mutations, and serrated-pathway biology, whereas left-sided CRC more commonly exhibits chromosomal instability, *APC* alteration, *TP53* mutation, and aneuploidy [15,17].

The mechanism of this location bias is not yet fully resolved, and direct proof that embryologic origin, vascular supply, or microbial gradients by themselves generate sex-specific right-sided risk remains limited. However, recent organoid and animal studies make it increasingly difficult to dismiss the signal as purely descriptive. Human normal-colon organoids show sex- and location-dependent transcriptional responses to calcium, indicating that epithelial programs already differ by colon site and sex before overt malignancy [18]. In AOM/DSS-based mouse models, estradiol restrains inflammatory signaling and tumor development, and sex-specific inflammatory responses correlate with ERβ and macrophage-associated cytokine programs [19,20]. These models do not yet prove a single mechanism for women-biased right-sided CRC, but they support the plausibility of site-specific sex effects arising from differences in epithelial responsiveness, inflammation, and microenvironmental gradients.

Sex intersects with this location gradient in clinically meaningful ways. Multivariable analyses have demonstrated location-dependent sex differences in programmed death-ligand 1 (PD-L1) expression, mismatch repair or microsatellite instability status, and epidermal growth factor receptor (EGFR) expression. In one study, women with proximal CRC were enriched for dMMR/MSI-H and high EGFR expression, whereas men with proximal disease showed an inverse association with PD-L1 expression [10]. More recent work suggests that right-sided CRC in women represents a particularly distinctive molecular niche: women with right-sided tumors were more likely to harbor a *BRAF* V600E-mutated/MSI-H combination and showed higher PD-L1 messenger RNA expression and nuclear factor erythroid 2-related factor 2 (NRF2) protein expression, together with lower NRF2 promoter methylation, than both men and women with left-sided disease [10,11].

These observations argue against treating sex and sidedness as competing variables. In CRC, the two often need to be modeled together because sidedness appears to expose, rather than obscure, important sex-linked biology. This is also the clearest setting in which an interaction model can be stated explicitly: the prognostic or biomarker meaning of sex differs according to whether the tumor is right-sided and whether that right-sided tumor is dMMR/MSI-H or *BRAF*-associated.

Sex-associated genomic architecture also extends beyond anatomical site. Women show higher frequencies of CIMP-high and *PIK3CA*-mutated disease in selected cohorts, and multiple datasets indicate enrichment of *BRAF*-associated and MSI-related tumors in women, particularly in proximal disease [10,11,21]. By contrast, one of the most compelling men-linked mechanisms identified to date is the *KRAS*–*STAT4*–*KDM5D* axis. In *KRAS*-mutant colon cancer, the Y-chromosome-encoded histone demethylase *KDM5D* is upregulated through *STAT4*-mediated transcription, promoting loss of epithelial barrier integrity, increased metastatic potential, and impaired tumor immune recognition [16]. At present, this axis should be viewed as a compelling but still incompletely validated model rather than as a definitive explanation of male CRC biology. Its plausibility is strengthened by independent evidence linking male *KRAS*-mutant CRC to suppressed ferroptosis programs and by emerging studies showing *KRAS*-associated cetuximab-resistance states and sex-linked *RAS*-associated recurrence patterns in rectal cancer [22,23,24]. However, direct multi-cohort validation of *KDM5D*-dependent effects remains a priority.

The same principle is apparent in younger patients. In a multi-ethnic AACR GENIE analysis of non-hypermutated EOCRC, *EP300* mutation was more frequent in younger men and substantially less frequent in younger women, while *KRAS*, *AXIN2*, *WRN*, *BRAF*, and additional genes also differed by sex [25]. A separate analysis integrating mutation patterns into a prognostic model further suggested that the clinical meaning of recurrent CRC mutations is itself sex-modulated rather than sex-neutral [26].

Metabolic studies reinforce the same contextual interpretation. Right-sided tumors in women display a nutrient-depleted phenotype with increased asparagine synthesis, and asparagine synthetase (ASNS)-related metabolic signatures have been linked to poorer survival in women [27,28]. In contrast, *KRAS*-mutant tumors in men have been associated with reduced ferroptotic activity and glutathione- and iron-handling programs linked to adverse outcome [22]. These are not simply parallel male and female metabolic signatures; they are genotype-aware and site-aware subphenotypes that support a contextual model of sex biology in CRC. These high-confidence sex-associated contexts are summarized in Table 1.

## 4. Estrogen Signaling and Epigenetic Circuitry: Still Important, but No Longer Sufficient

The earlier version of this review placed estrogen and epigenetics at the conceptual center of CRC sex-related biology. That emphasis captured an important part of the field, but as a stand-alone organizing framework it was too broad. Estrogen signaling is clearly relevant to CRC; however, its explanatory value increases when linked to specific receptors, transcriptional states, and disease contexts rather than invoked as a general protective umbrella. Recent data further support a receptor-specific interpretation of estrogen biology while underscoring that ER-associated signaling alone cannot account for the full spectrum of sex-related heterogeneity in CRC.

ERβ remains the most biologically persuasive node in this literature. It is the predominant estrogen receptor in normal colonic epithelium, is frequently reduced during tumor progression, and exerts anti-inflammatory and antitumor effects in colon cancer models [37,38,39,40]. ERβ can alter the microRNA repertoire of CRC cells, restrain metastasis through the miR-205–PROX1 axis, and associate with distinct tumor methylation programs [38,39,40]. Importantly, the human tissue literature is broader than we previously conveyed. In a prospective CRC cohort with 1101 tumors, loss of ERβ expression was associated with more advanced stage and poorer survival [41]. In female CRC cohorts, higher ERβ expression correlated with better overall and disease-free survival, and combined ERβ-high/ERα-negative expression identified a more favorable prognostic state [42,43]. A smaller paraffin-tissue series reported weaker prognostic association, underscoring that receptor isoform, cohort composition, and assay design matter [44]. Taken together, these studies support the interpretation that loss of ERβ function is one mechanism by which colonic epithelium may lose a sex-linked protective program during tumorigenesis.

At the same time, the estrogen story is more nuanced than a simple protective model in women. GPER can mediate differential proliferative and migratory effects under normoxic and hypoxic conditions, and epigenetic downregulation of GPER has been described as a tumor-suppressive event in CRC [45,46]. More recent work further suggests that GPER-related effects may themselves differ by sex, implying that receptor balance, oxygen tension, and molecular background all influence how estrogenic signaling is interpreted by tumor cells [47]. Preclinical AOM/DSS studies likewise show that estradiol, ERβ, and inflammatory polarization are linked in a sex-dependent manner [19,20].

A particularly important update is that sex-hormone signaling should now be connected to immunity rather than discussed only through proliferation or apoptosis. Intestinal ERβ has been shown to modulate the murine colon tumor immune microenvironment, linking classical hormone biology to checkpoint-relevant tumor ecology [48]. This bridge matters because it places estrogen-related biology within the same conceptual framework as sidedness, molecular subtype, and immune context, which is where its translational value becomes most visible. Conversely, the insufficiency of a receptor-only explanation becomes obvious when human sex effects persist in settings where ER status, microbiota, mutational subtype, and immune architecture do not move in parallel. This is why ER signaling is best interpreted as one mechanistic layer within a broader interaction framework rather than as a universal master explanation. Clinically, ER-related findings do not align uniformly with sidedness, KRAS/BRAF context, MMR/MSI status, or immune state across available cohorts, which is precisely why receptor biology alone cannot serve as a comprehensive explanatory framework for sex-related CRC heterogeneity.

The same logic applies to biomarker development. Machine-learning analyses have identified sex-contingent prognostic candidates in CRC [49]. Tissue-based and liquid-biopsy studies also report sex-related microRNA differences [50,51]. In parallel, epigenetic studies indicate that both background colonic methylation and cancer-associated methylation biomarkers can vary by anatomical site, sex, and age, with potential consequences for risk modeling and assay performance [52,53,54,55]. Taken together, these data suggest that sex-linked epigenetic variation is currently more informative for biomarker performance and assay interpretation than as a stand-alone explanation of CRC pathogenesis. Current evidence therefore supports sex-linked epigenetic variation more strongly as a modifier of biomarker context than as an independent driver framework for CRC pathogenesis.

## 5. Microbiota as an Ecological Amplifier of Sex Effects

Microbial ecology remains one of the most plausible routes through which sex, diet, inflammation, and environmental exposure converge in CRC. At the same time, microbiota research in this field is particularly vulnerable to overinterpretation. Heterogeneity arises not only from geography and diet, but also from whether studies use stool, mucosal, tumor-adjacent, or tumor-core specimens; whether profiling relies on 16S sequencing, shotgun metagenomics, metatranscriptomics, or metabolomics; and whether cohorts differ in bowel preparation, antibiotic exposure, body composition, tumor site, treatment status, or definition of control samples [56,57,58]. Recent location-aware multi-omics work further reinforces that host-microbe associations differ materially between right-sided and left-sided CRC, which is precisely why sex effects should not be interpreted without anatomic context [59]. These design differences likely contribute to the poor cross-cohort reproducibility of many purported sex-associated taxa and argue against treating any single microbiome signature as a universal sex marker in CRC without harmonized validation.

The most defensible position is therefore not that the microbiota explains CRC sex differences in general, but that it can amplify sex-linked risk or progression in selected biological settings. Human cohort studies have reported sex-associated differences in microbial richness, discriminatory taxa, and metabolite patterns in CRC, although the direction and magnitude of these differences vary across cohorts [60,61,62,63]. Experimental work provides more direct mechanistic support: men-biased microbiota and their metabolites can aggravate colorectal tumorigenesis, whereas specific bacterial species such as *Carnobacterium maltaromaticum* may suppress tumor development in a women-biased, vitamin D receptor-dependent manner [64,65].

Microbial effects are also anatomically contextual. Multi-omics analyses of right-sided and left-sided colon cancer have identified distinct microbe–metabolite–host networks, while bile-acid profiling has revealed sex-specific distributions associated with prognosis [59,66]. These findings are important because they align with the broader conclusion of this review: sex effects in CRC become most interpretable when layered onto anatomical site and metabolic state rather than analyzed in isolation.

**Figure 2:**
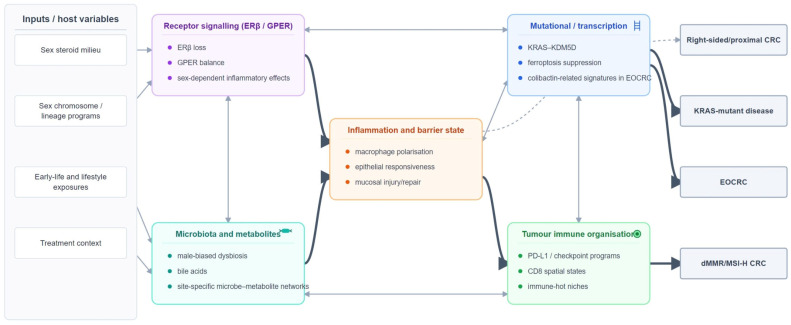
Mechanistic layers through which sex may modify CRC biology in selected contexts. The schematic integrates hormone signaling, gut microbiota, metabolic state, mutational processes, and tumor immune organization, emphasizing cross-talk and feedback rather than linear causation. The strongest evidence currently links these layers to right-sided CRC, dMMR/MSI-H disease, KRAS-mutant disease, and EOCRC.

The sex hormone–gut microbiome axis remains a useful conceptual framework, but it should be applied with caution [56]. The concept of the microgenderome describes bidirectional interactions among the microbiota, hormones, immunity, and disease susceptibility [58]. Experimental models further show that orchiectomy, testosterone administration, and estradiol exposure can reshape the gut microbiome during colorectal carcinogenesis, with downstream consequences for tumor development and immune tone [67,68]. This ecological framing is especially relevant to EOCRC and mutational-process research: sex-differential early-life exposures could plausibly influence colibactin-producing taxa, bile-acid biology, mucosal inflammation, or epithelial repair capacity, even though direct sex-dependent evidence for these initiation pathways is still sparse. The cross-talk among hormone signaling, microbiota, metabolic state, and immune ecology is integrated schematically in Figure 2. A more explicit testable model is that sex-dependent hormonal tone and immune-metabolic maturation influence mucosal barrier integrity, microbial persistence, and bile-acid ecology early in life, thereby affecting whether genotoxic communities such as colibactin-producing bacteria leave durable mutational footprints in susceptible epithelium.

## 6. Immune Microenvironment as the Central Translational Bridge

If one domain most directly links sex biology to clinical interpretation in CRC, it is the tumor immune microenvironment. CRC progression is strongly shaped by reciprocal interactions among malignant cells, stromal architecture, innate and adaptive immune compartments, and treatment-induced remodeling. Contemporary syntheses of the CRC immune microenvironment emphasize that immune evasion, immunosuppression, and therapeutic immune reprogramming are central to prognosis and to the selective success of immunotherapy, particularly in dMMR/MSI-H disease [69,70].

Quantification of CD8+ T-cell populations deserves more explicit attention because large multiplex immunofluorescence analyses of stage II–III CRC have shown that intratumoral CD8+ and FoxP3+ cell states carry independent prognostic information, and that immune context becomes more informative when quantified spatially rather than by crude density alone [71]. More granular multiplex studies further show that activated tissue-resident CD8+CD103+CD39+ cells are especially prognostic in left-sided “immune-hot” CRC, whereas terminally exhausted CD8+ states mark a distinct, genomically associated immune ecology in CRC [72,73].

Sex clearly matters in this space, but not through a simplistic rule that women always mount stronger antitumor immunity or that men are uniformly more immunosuppressed. Instead, the available evidence points to context dependence. In metastatic CRC models, men and women differ in cytokine patterns and in the balance of innate and adaptive immune compartments [69]. In right-sided human CRC, women tumors can show stronger PD-L1 and NRF2-related programs [11]. In hormone-linked preclinical systems, ERβ can reshape the colon tumor immune microenvironment [48]. Recent tumor-intrinsic work also shows that cell-cycle regulators such as LRRC19 can couple tumor suppression to immune microenvironment remodeling, illustrating the type of integrative biology needed for future sex-aware studies [74]. Taken together, these data support a model in which sex influences immune organization and checkpoint biology within specific molecular niches rather than across all CRCs equally.

This is precisely why the immune microenvironment functions as the central translational bridge in the present review. It provides a mechanistic explanation for why sex may be clinically silent in one CRC subgroup yet highly informative in another. A sex signal diluted in an unselected cohort may become obvious in right-sided immune-active tumors, in dMMR/MSI-H or *BRAF*-mutated metastatic CRC, or in younger patients with distinct mutational processes. Without an immune-context framework, sex differences remain largely descriptive; with it, they become biologically interpretable and potentially clinically actionable.

## 7. Biomarkers and Therapeutic Implications

The literature on sex-aware biomarkers in CRC is expanding, but its translational value depends on methodological discipline. Machine-learning analyses of paired normal and tumor tissues have identified men-biased and women-biased prognostic candidates such as ESM1, GUCA2A, VWA2, CLDN1, and FUT1 [49]. Other studies have reported sex-dependent microRNA patterns in tumor tissue and liquid biopsy [50,51]. Epigenetic analyses further indicate that methylation landscapes in normal colon and CRC can vary with anatomical location, age, and sex, which may influence the performance and interpretation of methylation-based assays [52,53,54,55].

The practical question is therefore not whether a biomarker differs between women and men in isolation. The more relevant question is whether incorporating sex materially improves discrimination, calibration, or treatment stratification once tumor site, molecular subtype, and immune context are already known. That is the threshold at which sex-aware biomarker work becomes clinically meaningful rather than merely descriptive. Accordingly, future biomarker studies should prespecify sex interaction testing, report model performance before and after adding sex, and annotate biospecimens with tumor sidedness, MMR/MSI status, *BRAF*/*KRAS* context, and treatment exposure. These requirements are more actionable than generic calls to “consider sex” and move the field toward deployable biomarker models. At minimum, prospective biomarker studies should prespecify sex as an interaction variable rather than merely a baseline covariate and should report discrimination and calibration before and after sex is added to the model.

Therapeutic data point in the same direction. In *BRAF*-mutated metastatic CRC, primary tumor sidedness and sex both influence prognosis and the activity of anti-epidermal growth factor receptor therapy, indicating that sex may interact with established clinical stratifiers rather than supersede them [12]. The FIRE-3 subgroup analysis likewise suggests that the efficacy of cetuximab plus FOLFIRI in *RAS*/*BRAF* wild-type metastatic CRC is associated with sex and primary tumor sidedness, supporting the inclusion of sex in the design and reporting of future trials [30]. In parallel, *KRAS*-mutant resistance biology is increasingly linked to male-associated transcriptional programs and cetuximab resistance states [16,22,23].

Immune-checkpoint-treated CRC offers an even clearer example of context-dependent sex effects. In MSI-H metastatic CRC with *BRAF* V600E mutation, outcomes after immune checkpoint inhibition differed according to the interaction between sex and *BRAF* context rather than according to sex alone. Men treated with anti-PD-(L)1 monotherapy had the poorest outcomes, whereas regimens containing anti-CTLA-4 appeared to attenuate this disadvantage; women, by contrast, showed a higher frequency of any-grade immune-related adverse events [29]. These results are hypothesis-generating rather than practice-changing, but they illustrate why sex should be prespecified in efficacy and toxicity analyses instead of being relegated to a post hoc demographic descriptor.

Accordingly, sex should not yet be used as a stand-alone treatment selector in CRC. The current evidence base is not strong enough for that. What the available literature does justify is a more rigorous analytical framework in which sex is modeled as an interaction term in biomarker studies, translational drug-response analyses, and prospective trial stratification—especially for right-sided disease, dMMR/MSI-H CRC, *BRAF*-mutant metastatic CRC, and anti-EGFR-treated populations. Protocol templates, translational substudies, and regulatory reporting standards should at minimum require sex-disaggregated efficacy and toxicity reporting in these high-yield settings. In practice, trials in these high-yield settings should prespecify sex-by-treatment interaction testing in the statistical analysis plan, provide sex-stratified efficacy and toxicity tables, and bank biospecimens with linked sidedness/MMR/MSI/BRAF/KRAS annotation for correlative analyses.

## 8. Early-Onset Colorectal Cancer as a High-Priority Context

Among emerging CRC contexts, EOCRC is arguably the most informative setting for future sex-aware research. The latest United States statistics show that CRC incidence and mortality are increasing in adults younger than 65 years, with particularly concerning trends in those aged 20–49 years; global registry analyses similarly show that EOCRC incidence is rising in many countries, often more rapidly than in older adults [2,31]. This epidemiological shift alone justifies increased attention to EOCRC. More importantly, accumulating evidence indicates that EOCRC is biologically distinct rather than simply a younger presentation of conventional later-onset CRC.

Large-scale sequencing studies support that view. A 1209-patient next-generation sequencing analysis identified a distinctive molecular fingerprint in EOCRC [32]. An international multicohort study further showed that genomic mutational patterns differ between EOCRC and later-onset CRC according to hypermutation context [33]. Together, these findings make two points clear: EOCRC is not biologically uniform, and age of onset interacts with mutational architecture in ways that standard pooled CRC analyses can obscure.

Recent work on mutational processes has widened the etiologic horizon. In a Nature study of 981 CRC genomes from 11 countries, the colibactin-related mutational signatures SBS88 and ID18 were enriched in EOCRC, were especially common in individuals diagnosed before 40 years of age, and were linked to early *APC* driver events [34]. These data do not prove a sex mechanism by themselves, but they strongly support models that integrate age, ecological exposure, and host biology when evaluating CRC initiation and progression. A plausible next step is to test whether sex-dependent differences in hormones, barrier function, inflammatory tone, or microbiota composition alter the likelihood that early-life bacterial genotoxins leave persistent mutational footprints. This question is not yet answered, but EOCRC is precisely the setting in which it becomes experimentally and clinically tractable. This places mutational-process work within the same host-microbe-sex framework discussed above rather than treating SBS88/ID18 as age-only phenomena.

Sex remains underdeveloped in EOCRC research, but it is already testable. Younger-age sequencing data show sex-differential mutation patterns [25], and recent educational and translational reviews emphasize the need for EOCRC-specific molecular profiling and study design [35]. Population-level analyses have also begun to show sex-related differences in EOCRC survival and treatment pathways, underscoring that biologic sex and gender-shaped care processes may both contribute to observed disparities [14,36]. EOCRC should therefore be treated as a high-priority context for sex-aware CRC biology: not because current evidence is already definitive, but because rising incidence, distinct mutational processes, and major unmet clinical need together make EOCRC the most informative setting in which to validate sex-aware models. An evidence-and-gap overview of sex-aware EOCRC research is provided in Table 2. For this reason, sex-aware EOCRC research should integrate biologic sampling with diagnosis-to-treatment pathway data rather than assuming that all observed disparities are tumor intrinsic.

## 9. Conclusions and Future Directions

The most useful contemporary view of sex-related heterogeneity in CRC is not that male and female CRCs constitute two separate diseases, nor that estrogen or the microbiota alone explains the observed disparities. Rather, sex acts as a contextual modifier whose biological and clinical relevance becomes most visible when layered onto tumor sidedness, molecular subtype, immune ecology, and age at onset. Within this framework, women are enriched for selected right-sided, MSI-related, and *BRAF*-associated disease states, whereas men carry a higher overall burden of CRC and show distinctive programs such as *KRAS*–*KDM5D*-linked metastatic and immune-evasive biology. Estrogen signaling, ERβ loss, GPER activity, and sex-linked microbial ecology remain important, but their translational significance lies in how they shape specific CRC states rather than in their ability to explain all sex-related differences at once.

Three testable propositions emerge from this revised framework. First, the strongest clinical value of sex in CRC is likely to come from interaction models—especially sex × sidedness × dMMR/MSI-H/*BRAF* context—rather than from sex-alone subgrouping. Second, *KRAS*-mutant disease remains the leading setting in which male-associated metastatic and therapy-resistance programs may prove mechanistically and therapeutically relevant, but this requires external validation before clinical use. Third, EOCRC is the most informative setting in which to determine whether sex improves etiologic inference, biomarker calibration, and prospective trial design.

Several priorities follow from this framework. Future studies should separate sex from gender at the design stage and should specify whether they are testing biological sex, gender-related behavior, or both, because risk exposure, screening uptake, diagnostic interval, treatment experience, and survivorship are not interchangeable constructs [4,8,13,14]. In EOCRC particularly, sex-aware designs should also capture time to diagnosis and time to treatment, because care-pathway disparities can otherwise be misread as purely biologic effects [14,36]. Sex should also be analyzed through interactions rather than through simple frequency comparisons. The most informative models will test sex together with tumor sidedness, dMMR/MSI-H status, BRAF or KRAS context, immune state, and age at onset. Without this step, sex-associated signals will continue to appear fragmented and inconsistently reproducible across cohorts.

Biomarker development should move beyond descriptive subgrouping. Liquid biopsy, methylation, microRNA, spatial transcriptomic, and single-cell platforms should ask whether sex improves model performance in defined clinical contexts, not merely whether a biomarker differs by sex in the abstract [49,50,51,52,53,54,55,70]. Translational work should prioritize the settings in which sex already appears biologically non-random: right-sided CRC, dMMR/MSI-H disease, *BRAF*-associated metastatic CRC, male *KRAS*-linked metastatic programs, and EOCRC. These are more likely to yield clinically useful findings than unselected all-stage CRC cohorts. Therapeutic studies should routinely report sex-disaggregated efficacy and toxicity data and prespecify how sex will be handled analytically. This is particularly important for anti-EGFR strategies and immune checkpoint blockade, where emerging evidence suggests that sex modifies treatment interpretation but does not yet justify sex-based prescribing decisions on its own [29,30]. At the level of research practice, protocol templates, translational substudies, and biobanking pipelines should incorporate mandatory sex annotation, interaction testing, and sex-stratified reporting in the high-yield settings identified in this review. Near-term priorities should now be stated more explicitly: prospective cohorts should prespecify sex × sidedness × dMMR/MSI-H/BRAF interactions; multicenter *KRAS*-mutant studies should validate *KDM5D*-, ferroptosis-, and treatment-resistance signatures with matched outcome data; and EOCRC programs should integrate mutational signatures, early-life exposure metrics, and microbiome profiling from enrollment onward.

Accordingly, the future value of sex-aware CRC research will lie less in repeatedly showing that differences exist and more in defining where those differences alter biomarker interpretation, prognostic modeling, therapeutic benefit, toxicity, and etiologic inference. A sex-aware, subtype-aware, and location-aware framework is therefore increasingly necessary for a biologically mature and clinically useful understanding of CRC. As the evidence matures, guideline panels and trial-reporting standards should move from optional subgroup description toward prespecified sex-aware interaction analyses in the specific CRC contexts identified here.

## Figures and Tables

**Table 1:** Clinical and molecular contexts in which sex currently provides the strongest evidence in colorectal cancer.

Clinical Context	What is Currently Sex-Contingent?	Dominant Evidence Base	Actionable Implication Now	Key References
Right-sided/proximal CRC	Women are enriched for proximal disease, and in selected right-sided cohorts sex also tracks with dMMR/MSI-H, PD-L1, NRF2, and *BRAF*-associated features.	Primarily retrospective human tissue and cohort studies, supported by site- and sex-dependent preclinical transcriptional/inflammatory models.	Do not pool right- and left-sided CRC when testing sex effects. Prespecify sex × sidedness interaction terms in biomarker, prognosis, and translational analyses.	[6,7,8,9,10,11,15,17,18,19,20]
dMMR/MSI-H CRC	Sex effects become most visible after tumor site and BRAF context are layered; women enrichment is strongest in proximal MSI-related disease rather than in unselected MSI cohorts.	Retrospective pathology and metastatic clinical outcome analyses.	Report efficacy and toxicity separately by sex inside dMMR/MSI-H cohorts and test sex × *BRAF* × treatment interactions; do not use sex alone as an immunotherapy selector.	[10,11,12,17,29]
*BRAF*-mutated metastatic CRC	Women are overrepresented in selected right-sided/*BRAF*-associated contexts, while sex and primary sidedness jointly influence prognosis and anti-EGFR activity.	Pooled metastatic clinical cohorts and subgroup analyses.	In anti-EGFR and *BRAF*-focused datasets, model sex jointly with primary tumor sidedness and molecular context instead of treating sex as a post hoc descriptor.	[11,12,17,29,30]
*KRAS*-mutant CRC	Men-linked programs are most evident in defined *KRAS*-mutant settings rather than across CRC as a whole.	Mechanistic preclinical work plus clinically annotated molecular cohorts.	Prospectively validate *KDM5D*-, ferroptosis-, and resistance-related signatures in sex-stratified *KRAS*-mutant CRC; sex is not yet ready for treatment selection.	[16,22,23,24]
Early-onset colorectal cancer (EOCRC)	Sex differences are detectable but remain underdeveloped; some younger-age cohorts show sex-differential mutation patterns and potentially sex-modulated survival signals.	Large sequencing cohorts, registry analyses, and emerging EOCRC-specific outcome studies.	Build sex-disaggregated EOCRC registries with harmonized site, subtype, microbiome/exposure, and treatment annotation; use EOCRC as a high-yield validation setting.	[25,26,31,32,33,34,35,36]

Abbreviations: CRC, colorectal cancer; dMMR, deficient mismatch repair; MSI-H, microsatellite instability-high; EOCRC, early-onset colorectal cancer; EGFR, epidermal growth factor receptor.

**Table 2:** Why early-onset colorectal cancer is a high-priority setting for sex-aware investigation.

Domain	Current Evidence	Why this Remains High Priority Despite Limitations	Recommended Next Step	Key References
Epidemiology	Incidence is rising in many countries, particularly among adults aged < 50 years; EOCRC can no longer be interpreted as a rare extension of later-onset disease.	A rapidly increasing disease burden makes even incomplete sex-aware evidence clinically important, because cohort design decisions made now will shape future inference.	Build sex-disaggregated, age-resolved population studies that integrate lifestyle, screening history, diagnostic interval, treatment timeliness, ancestry, and tumor biology.	[2,31]
Molecular profiling	Large sequencing cohorts show that EOCRC has a partly distinct molecular fingerprint and that some recurrent alterations differ by sex in younger patients.	Even when current cohorts are underpowered for interaction testing, the signal is strong enough to justify mandatory sex annotation in EOCRC omics studies.	Use larger multicenter cohorts with prespecified sex-by-age interaction analyses and harmonized site/subtype annotation.	[25,32,33]
Mutational processes and exposures	Genome-scale work links EOCRC in selected patients to colibactin-associated signatures and early APC driver events.	This is the clearest mechanistic entry point for integrating sex, microbiota, and early-life exposure in future work.	Test whether early-life microbial exposures, mutational signatures, and host sex jointly shape EOCRC initiation pathways.	[34,35,57,58]
Immune context and therapy	EOCRC may differ in immune organization and treatment response, but robust sex-aware immune datasets remain sparse.	Therapeutic and immune studies launched now can still incorporate sex prospectively rather than retrofitting it later.	Incorporate spatial immune profiling, transcriptomics, and treatment outcomes into EOCRC studies with mandatory sex reporting.	[35,71,72,73,74]
Clinical translation	EOCRC concentrates several features that make sex-aware modeling potentially informative: rising burden, distinct biology, and major unmet need in risk stratification. Emerging population studies also suggest sex-related differences in survival and treatment timeliness.	High priority does not mean evidence is already practice-changing; it means this is the setting where sex-aware study design is most likely to yield clinically useful results.	Prioritise prospective EOCRC registries and translational trials that treat sex as an interaction variable rather than a simple baseline covariate.	[2,14,25,31,32,33,34,35,36]

Abbreviations: EOCRC, early-onset colorectal cancer; APC, *adenomatous polyposis coli*.

## Data Availability

No new data were created or analyzed in this study. Data sharing is not applicable to this article.
